# Macules and verrucous lesions erupting in a pediatric transplant patient

**DOI:** 10.1002/hsr2.167

**Published:** 2020-07-02

**Authors:** Jacqueline S. Stevens, Vernon J. Forrester, Barrett J. Zlotoff

**Affiliations:** ^1^ School of Medicine University of Virginia Charlottesville Virginia; ^2^ Department of Dermatology University of Virginia Charlottesville Virginia

A 10‐year‐old male with history of heart transplant presented with rash resembling tinea versicolor and flat warts of 1‐year duration. Examination revealed hypopigmented macules with overlying fine scale from the neck to mid‐back (Figure 1A). Bilateral extremities exhibited several flat‐topped pink papules coalescing into plaques (Figure 1B). Laboratory evaluation revealed leukopenia and reduced T‐cell activity, consistent with immunosuppressive therapy. Biopsies from two different sites demonstrated keratinocytes with pale blue cytoplasm, multiple keratohyaline granules, and thickened granular layer (Figure 1C). Macules and verrucous lesions with these histopathologic findings are pathognomonic for epidermodysplasia verruciformis (EV). EV can be inherited (IEV) or acquired (AEV). In both forms, β‐type human papilloma virus (β‐HPV) drives skin lesions. AEV is reported in persons with HIV/AIDS and in solid organ transplant recipients on immunomodulatory medications.^1^, ^2^

**Figure 1:**
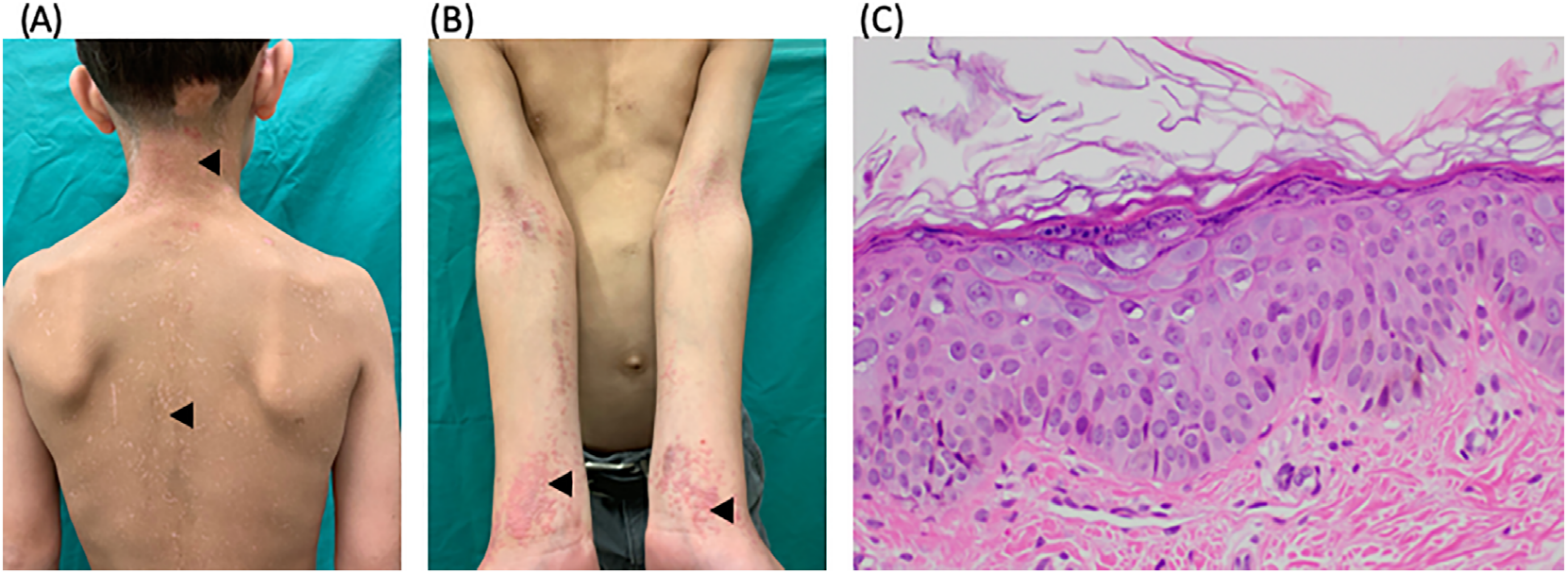
A, Hypopigmented to light pink macules, some with overlying fine scale (arrowheads); B, Flat‐topped pink papules coalescing into plaques (arrowheads); C, Representative hematoxylin and eosin (H&E) staining of the two biopsy specimens, ×40

In AEV, depressed cell‐mediated immunity results in increased susceptibility to otherwise nonpathogenic β‐HPV types, most commonly 5 and 8.^1^ While our patient's lesions did not appear in early childhood, it was important to rule out IEV as this would significantly affect management. Genetic testing for genes implicated in IEV was negative. Treatment for AEV is directed at reducing and/or switching immunosuppressive medication; our patient's immunosuppressive therapy was reduced, resulting in minimal rash improvement without evidence of transplant rejection. The patient has since tried multiple therapies including imiquimod and cidofovir without complete resolution. Many patients with IEV develop squamous cell carcinoma (SCC) by age 30, however, the risk is not as well characterized in AEV.^3^, ^4^ Further, determination of EV‐associated risk is complicated by known increased risk of malignancy, particularly SCC, in solid organ transplant recipients.^3^, ^4^ This patient highlights the unique challenge of treating AEV in pediatric transplant patients, which requires ruling out IEV, reducing/changing immunosuppressive medications, trial of therapies with varied reported efficacies, and counseling on increased risk of SCC.

## CONFLICT OF INTEREST

The authors declare no conflicts of interest.

## AUTHOR CONTRIBUTIONS

Conceptualization: Jacqueline S. Stevens, Vernon J. Forrester, Barrett J. Zlotoff

Writing – original draft preparation: Jacqueline S. Stevens

Writing – review and editing: Jacqueline S. Stevens, Vernon J. Forrester, Barrett J. Zlotoff

All authors have read and approved the final version of the manuscript.

The lead author (Jacqueline S. Stevens) and corresponding author (Vernon J. Forrester) had full access to all of the data in this study and take complete responsibility for the integrity of the data and the accuracy of the data analysis. All authors were involved in the care of the patient.
